# Shotgun Metagenomic Analysis Reveals Taxonomic and Functional Transitions in the Salivary Microbiome During Periodontal Disease Progression

**DOI:** 10.1111/jcpe.70184

**Published:** 2026-08-09

**Authors:** Muhammed Manzoor, Jaakko Leskelä, Eija Könönen, Leo Lahti, Jukka Putaala, Pirkko J. Pussinen, Susanna Paju

**Affiliations:** ^1^ Department of Oral and Maxillofacial Diseases University of Helsinki Helsinki Finland; ^2^ School of Medicine, Institute of Dentistry University of Eastern Finland Kuopio Finland; ^3^ Institute of Dentistry University of Turku Turku Finland; ^4^ Department of Computing University of Turku Turku Finland; ^5^ Clinical Neurosciences, University of Helsinki and Department of Neurology Helsinki University Hospital Helsinki Finland

**Keywords:** gingivitis, microbial dysbiosis, multi‐kingdom interactions, oral biomarkers, oral microbiome, periodontitis, saliva metagenomics

## Abstract

**Aim:**

To characterise multi‐kingdom salivary microbiome profiles across clinically defined periodontal states and identify stage‐specific taxonomic and functional alterations using shotgun metagenomic sequencing.

**Materials and Methods:**

In this cross‐sectional study, 204 adults (mean age 40.3 ± 7.6 years) from the SECRETO study (NCT01934725) underwent clinical and radiographic oral examinations and were classified into six periodontal groups: periodontal health, localised gingivitis, generalised gingivitis, gingivitis with pockets, mild periodontitis (Stages I–II) and severe periodontitis (Stages III–IV). Saliva samples were analysed using shotgun metagenomic sequencing to evaluate microbial diversity, taxonomic composition and functional pathways.

**Results:**

Beta diversity differed between periodontal health and the different disease states (Bray–Curtis: *p* = 0.049; Jaccard: *p* = 0.043). Gingivitis with pockets and severe periodontitis showed a significant enrichment of disease‐associated species *Porphyromonas gingivalis* , *Tannerella forsythia* , *Treponema denticola* , *Porphyromonas endodontalis* , *Fusobacterium nucleatum* and *Parvimonas micra* . Among non‐bacterial taxa, *Candida*, *Moineauvirus*, *Pyricularia* and *Roseolovirus* were the predominant genera. A composite metagenomic classifier showed high discriminative performance for gingivitis with pockets (AUC = 0.90; 95% CI: 0.770–1.000) and severe periodontitis (AUC = 0.865; 95% CI: 0.762–0.968).

**Conclusion:**

Salivary multi‐kingdom microbiome transitions closely reflect the progression of periodontal disease and provide promising biomarkers for identifying at‐risk individuals.

## 1 Introduction

Periodontal health progresses through a continuum that includes gingival health, gingivitis and periodontitis, with each stage characterised by distinct microbial changes (Iniesta et al. 2023). In periodontal health, the oral microbiome is typically diverse and largely composed of commensal species that maintain an ecological balance (Lenartova et al. 2021).

Gingivitis, a reversible inflammatory condition, invariably precedes periodontitis, although not all cases progress. As initial and early gingivitis lesions evolve, the condition may advance to an established lesion characterised by moderate to severe gingival inflammation, where biofilms extend deeper into the gingival sulcus and periodontal pockets (Cekici et al. 2014). Gingivitis is reversible with appropriate intervention, but if left untreated, it may progress to advanced, irreversible periodontal destruction. This transition is marked by a gradual increase in inflammatory burden and microbial dysbiosis (Kurgan and Kantarci 2018), ultimately leading to tissue destruction, as observed in periodontitis.

Periodontitis progression is influenced by multiple risk factors, with microbial components playing a central role alongside pre‐existing periodontal conditions such as deep probing depths and plaque‐retentive areas (Genco and Sanz 2020). Bacterial colonisation, multiplication and invasion of the gingival sulcus are key steps in disease initiation (Tribble and Lamont 2010), and host‐related factors critically influence its progression. In periodontitis, the microbial balance is disrupted, leading to the enrichment of classical pathogens, such as *Porphyromonas gingivalis* , *Treponema denticola* and *Tannerella forsythia* (Socransky et al. 1998). These pathogens form complex biofilms (Aruni et al. 2015), trigger destructive host inflammatory responses and contribute to the loss of beneficial microbes and tissue destruction (Mohanty et al. 2019).

The subgingival microbiome residing within gingival sulci and periodontal pockets serves as a key indicator of periodontal status and differs significantly from that of other oral sites (Aas et al. 2005). Saliva is a composite of microbial populations that are shed from various intraoral surfaces. Nevertheless, salivary microbiota composition reflects periodontal conditions and correlates with clinical parameters (Jung et al. 2024). Many studies have used 16S rRNA sequencing to investigate periodontitis‐associated microbiomes, mainly at the subgingival and supragingival sites (Abusleme et al. 2013; Park et al. 2015; Pozhitkov et al. 2015; Nemoto et al. 2021; Lee et al. 2026), whereas shotgun metagenomic profiling of saliva across the full spectrum of periodontal disease is scarce and warrants further investigation.

Although we have previously reported distinct microbial differences in early periodontitis using saliva metagenomics (Manzoor et al. 2024a), shotgun analyses across disease stages are still lacking. Therefore, this study aimed to characterise multi‐kingdom salivary microbiome profiles from periodontal health to severe periodontitis using shotgun metagenomics, examining both species‐level and functional shifts across disease stages.

## 2 Materials and Methods

### 2.1 Study Population

The present study, SECRETO Oral (Leskelä et al. 2024), is a sub‐study of the international, multi‐centre SECRETO case–control study (NCT01934725) (Putaala et al. 2017). For this study, we included 204 participants with varying periodontal health and disease states but excluded individuals who met any of the following criteria: antibiotic use within 1 month before saliva sampling, lack of knowledge of antibiotic use, missing information on periodontitis and participants who had professional dental cleaning less than 1 month prior to saliva sampling.

### 2.2 Clinical and Radiographic Oral Examinations

The clinical parameters of the study participants were assessed by a single periodontist (S.P.) between April 2014 and February 2020. Panoramic tomography images were obtained for all participants.

Full periodontal assessment was performed including probing pocket depth (PPD) and bleeding on probing (BOP) at six sites per tooth. Gingival recession, suppuration and tooth mobility were also recorded. Clinical furcation involvement was evaluated using Hamp's classifications (Hamp et al. 1975). Periodontitis stage and grade were determined on the basis of radiographic bone loss assessment (Tonetti et al. 2018).

After clinical and radiological oral examinations, the study subjects were divided into six groups based on their periodontal status according to the modified version of the 2018 Classification of Periodontal and Peri‐Implant Diseases and Conditions. Subjects with no radiographic alveolar bone loss were categorised into four groups: periodontal health (H), with no clinical signs of periodontitis and < 20% BOP; localised gingivitis (LG) defined as 20%–30% BOP; generalised gingivitis (GG), defined as > 30% BOP. Subjects in the H, LG and GG groups may have had incidental PPDs ≥ 4 mm, such as pseudo‐pockets at distal sites of erupted but infra‐positioned wisdom teeth, pockets at the distal sites of the second molars next to impacted or partially erupted wisdom teeth or pockets next to overhanging dental restorations and crowns. The gingivitis with pockets (GP) group consisted of subjects with more than eight sites with PPD ≥ 4 mm and BOP > 30% but no alveolar bone loss. Subjects with radiographic alveolar bone loss were divided into two groups. Mild periodontitis (MP) was defined as bone loss at stages I–II, and severe periodontitis (SP) was defined as bone loss at stages III–IV (Chapple et al. 2018; Papapanou et al. 2018). PPDs around the wisdom teeth with partial eruption or pericoronitis were excluded from the total number of pockets from all study subjects. Peri‐implant infections did not affect the periodontal diagnosis group.

### 2.3 Saliva Sampling and Metagenomic Sequencing

Sample processing and DNA extraction are described in detail elsewhere (Manzoor et al. 2024b, 2025). Briefly, genomic DNA was extracted and metagenomic sequencing was performed on an Illumina NovaSeq 6000. Taxonomy was assigned using Kraken2 (v.2.1.2) (Wood et al. 2019) and Bracken (v.2.7.0) (Lu et al. 2017).

### 2.4 Statistical Analysis

Statistical analyses were conducted using R (v.4.2.2). Group differences were assessed using ANOVA or Kruskal–Wallis tests for continuous variables, and *χ* ^2^ or Fisher's exact tests for categorical variables. Microbial alpha diversity was assessed using the Shannon and Inverse Simpson indices using the vegan R package (v. 2.6.4). Permutational multivariate analysis of variance (PERMANOVA) was conducted using Bray–Curtis dissimilarity and Jaccard dissimilarity to assess differences in microbial community composition across periodontal groups, adjusting for age, sex, smoking history and recent antibiotic use in the preceding 1–3 months. Principal coordinates analysis (PCoA) was applied to both dissimilarity matrices to visualise species‐level beta diversity differences among groups. Differential abundance analysis was performed using linear discriminant analysis effect size (LEfSe; Segata et al. 2011).

To explore the characteristics of oral microbiomes in GP and SP patients compared with H subjects, a logistic regression model was fitted with five‐fold cross‐validation to identify discriminant species based on the area under the receiver operating characteristic (ROC) curve (AUC). For network analysis, a SparCC‐based co‐occurrence network was built with SpiecEasi using the *microeco* (v. 1.7.1) R package. Network visualisation was performed with Gephi (v0.9.7) (Bastian et al. 2009).

Functional profiling of shotgun sequencing data was performed with HUMAnN3 (Beghini et al. 2021) and the differential abundance of functional features was assessed using MaAsLin 2 (Mallick et al. 2021).

See Supporting Information for further details.

## 3 Results

### 3.1 Clinical Characteristics of Participants

The clinical characteristics of the study participants are summarised in Table 1. The study involved 204 participants categorised into six groups: H (*n* = 15), LG (*n* = 34), GG (*n* = 41), GP (*n* = 43), MP (*n* = 35) and SP (*n* = 36). The overall proportion of males was 61.3%, with significant differences between the groups (*p* < 0.001). The mean age of participants was 40.3 (SD 7.6) years, which differed significantly across groups (*p* = 0.002). Abdominal obesity and smoking varied significantly between the groups (*p* < 0.05). However, hypertension, diabetes and heavy alcohol use did not differ significantly between the groups.

**TABLE 1:** Characteristics of study subjects.

	H (*n* = 15)	LG (*n* = 34)	GG (*n* = 41)	GP (*n* = 43)	MP (*n* = 35)	SP (*n* = 36)	Total (*n* = 204)	*p*
Male sex	8 (53.3)	9 (26.5)	19 (46.3)	36 (83.7)	26 (74.3)	27 (75.0)	125 (61.3)	**< 0.001**
Mean age (SD), years	42.1 (8.8)	36.9 (6.9)	37.9 (9.3)	40.8 (7.3)	42.4 (6.1)	42.9 (5.9)	40.3 (7.6)	**0.002**
Abdominal obesity	3 (20)	10 (29.4)	11 (26.8)	32 (74.4)	18 (51.4)	24 (66.7)	98 (48.0)	**< 0.001**
Smoking, ever	10 (66.7)	11 (32.4)	15 (36.6)	21 (48.8)	13 (37.1)	29 (80.6)	99 (48.5)	**< 0.001**
Hypertension	2 (13.3)	7 (20.6)	8 (19.5)	9 (20.9)	7 (20.0)	13 (36.1)	46 (22.5)	0.415
Diabetes mellitus	1 (6.7)	1 (2.9)	1 (2.4)	1 (2.3)	3 (8.6)	2 (5.6)	9 (4.4)	0.742
Heavy alcohol use	2 (13.3)	3 (8.8)	6 (14.6)	9 (20.9)	1 (2.9)	9 (25)	30 (14.7)	0.099
History of stroke	9 (60.0)	10 (29.4)	16 (39.0)	26 (60.5)	17 (48.6)	24 (66.7)	102 (50.0)	**0.014**
Antibiotic use in the preceding 1–3 months	0	1 (2.9)	2 (4.9)	3 (7.0)	1 (2.9)	3 (8.3)	10 (4.9)	0.750
Illicit drug use in the preceding 12 months	0	3 (8.8)	4 (9.8)	1 (2.3)	0	1 (2.8)	9 (4.4)	0.198
Regular dentist check‐ups	12 (80)	22 (64.7)	27 (65.9)	19 (44.2)	24 (68.6)	17 (48.6)	121 (59.3)	**0.046**
Caries	5 (33.3)	10 (29.4)	18 (43.9)	24 (55.8)	14 (40.0)	20 (55.6)	91 (44.6)	0.146
Bleeding on probing, mean (SD)	16.68 (2.7)	24.7 (3.3)	44.25 (9.9)	51.7 (11.5)	36.9 (11.3)	48.4 (16.3)	40.0 (15.6)	**< 0.001**
Clinical furcations	0	2 (5.9)	1 (2.4)	8 (18.6)	2 (5.7)	24 (66.7)	37 (18.1)	**< 0.001**
Horizontal alveolar bone loss								
No bone loss	15 (100)	34 (100)	41 (100)	43 (100)	0	0	133 (65.2)	**< 0.001**
< 15	0	0	0	0	28 (80)	20 (55.6)	48 (23.5)	
15–33	0	0	0	0	7 (20)	12 (33.3)	19 (9.3)	
> 33	0	0	0	0	0	4 (11.1)	4 (2)	
No. of missing teeth								
0	14 (93.3)	27 (79.4)	34 (82.9)	36 (83.7)	25 (71.4)	16 (44.4)	152 (74.5)	**0.001**
1–2	0	2 (5.9)	5 (12.2)	5 (11.6)	6 (17.1)	7 (19.4)	25 (12.3)	
3–5	1 (6.7)	5 (14.7)	2 (4.9)	2 (4.7)	4 (11.4)	10 (27.8)	24 (11.8)	
> 5	0	0	0	0	0	3 (8.3)	3 (1.5)	
No. of periodontal pockets (≥ 4 mm)								
0–4	15 (100)	34 (100)	41 (100)	0	24 (68.6)	7 (19.4)	121 (59.3)	**< 0.001**
5–10	0	0	0	14 (32.5)	10 (28.5)	4 (11.1)	28 (13.7)	
> 10	0	0	0	29 (67.5)	1 (2.9)	25 (69.5)	55 (27.0)	
No. of periodontal pockets (≥ 6 mm)								
0–2	15 (100)	34 (100)	41 (100)	37 (86)	34 (97.1)	24 (66.7)	185 (90.7)	**< 0.001**
3–5	0	0	0	6 (14)	1 (2.9)	4 (11.1)	11 (5.4)	
> 5	0	0	0	0	0	8 (22.2)	8 (3.9)	

*Note:* Data are *n* (%) unless otherwise indicated. Abdominal obesity defined as waist‐to‐hip ratio; men ≥ 0.9, women ≥ 0.85.

Abbreviations: BOP: bleeding on probing; GG: generalised gingivitis; GP: gingivitis with pockets; H: periodontal health; LG: localised gingivitis; MP: mild periodontitis (Stage I/II); *n*: sample size (%); SD: standard deviation; SP: severe periodontitis (Stage III/IV).

The presence of dentine carious lesions did not significantly differ between the groups (*p* = 0.146). BOP and the number of missing teeth increased with disease severity (*p* < 0.001). Clinical furcation involvement was highest in SP (*p* < 0.001). The proportion of participants with periodontal pockets ≥ 4 mm and ≥ 6 mm also increased with disease severity (*p* < 0.001; Table 1).

### 3.2 Alpha and Beta Diversity Across Periodontal Conditions

Alpha diversity indices showed an increasing trend from H to GP, followed by a decline (Figure 1a; Table S1). Shannon index found a significant difference among the periodontal groups (*p* = 0.018), and Shannon diversity was higher in the GP group compared with the H group (*p* = 0.007). No other pairwise comparisons reached statistical significance (Table S2). There were no statistically significant differences in Inverse Simpson diversity across groups (*p* = 0.156).

**FIGURE 1:**
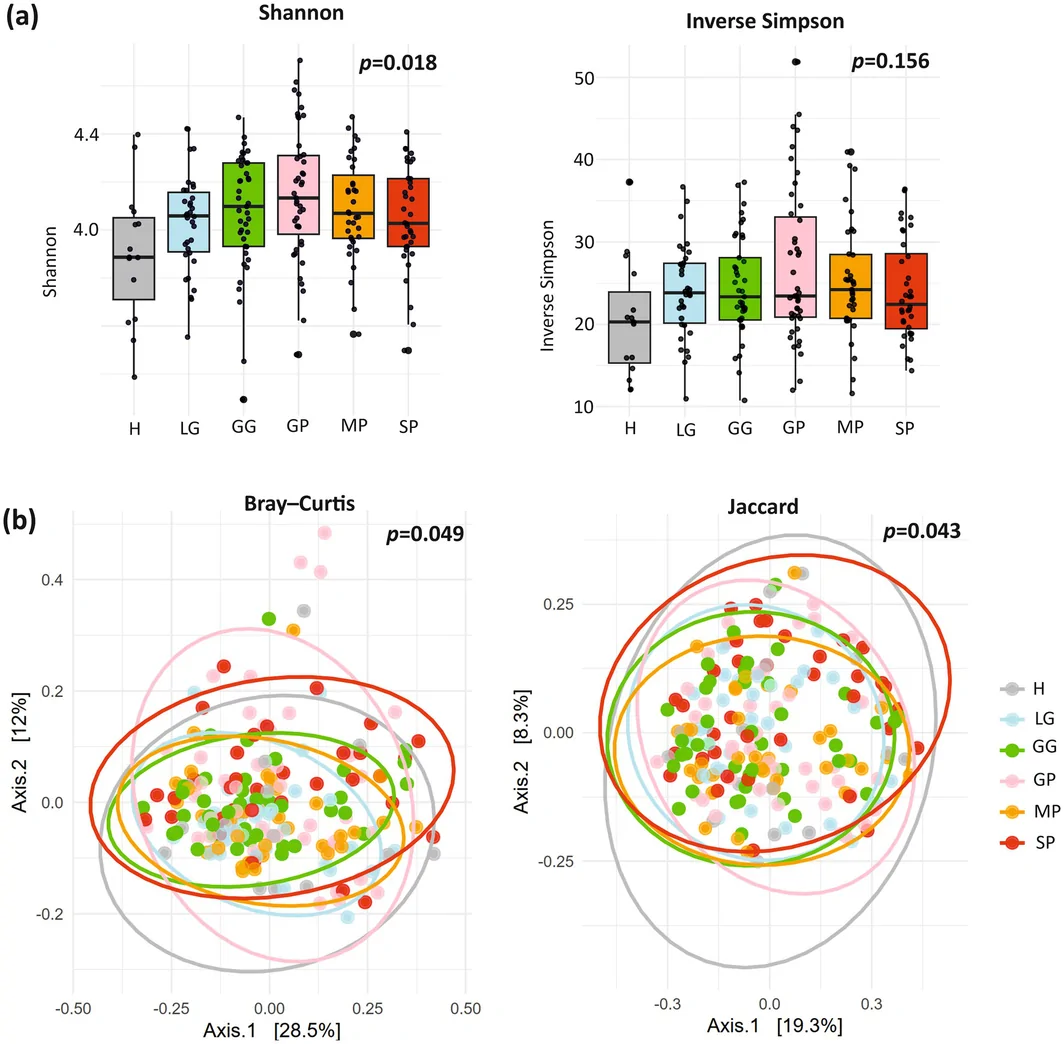
Oral microbiome diversity across periodontal health and disease states. (a) Alpha diversity, measured by Shannon and Inverse Simpson indices, across periodontal health and disease states. (b) Principal coordinates analysis (PCoA) plots illustrate beta diversity using Bray–Curtis and Jaccard dissimilarities at the species level across periodontal health and disease states. Ellipses represent the 95% confidence intervals for each group. GG, generalised gingivitis; GP, gingivitis with pockets; H, periodontal health; LG, localised gingivitis; MP, mild periodontitis (Stage I/II); SP, severe periodontitis (Stage III/IV).

PERMANOVA revealed significant differences in microbiome composition across the periodontal groups at the species level, based on both Bray–Curtis (*F* = 1.276, *R* ^2^ = 0.031, *p* = 0.049) and Jaccard (*F* = 1.277, *R* ^2^ = 0.031, *p* = 0.043) dissimilarities. These species‐level differences were also evident in the PCoA plots (Figure 1b). Pairwise comparisons revealed a significant difference in microbiome composition only between LG and GP (Bray–Curtis: *p* = 0.004 and Jaccard distances: *p* = 0.011). Additionally, Jaccard distances showed a significant difference between LG and SP (*p* = 0.037; Table S3). Adjusted PERMANOVA models (age, sex, smoking and antibiotic use) revealed that periodontal groups (Bray–Curtis: *p* = 0.048, Jaccard: *p* = 0.053) and smoking (Bray–Curtis: *p* = 0.042, Jaccard: *p* = 0.044) were significantly associated with microbiome composition at the species level (Table S4).

### 3.3 Taxonomic Composition of Periodontal Conditions

Phyla Bacillota, Actinomycetota, Bacteroidota, Pseudomonadota and Fusobacteriota predominated across periodontal health and disease states (Figures 2a and S1a). Bacillota, Candidatus Saccharibacteria, Spirochaetota, and Synergistota varied significantly with disease progression, with the latter two enriched in SP (*p* < 0.05). The Bacillota/Bacteroidota (Firmicutes/Bacteroidetes) ratio was highest in the SP group (*p* = 0.038). The relative abundance of Actinomycetota, Bacteroidota, Pseudomonadota and Fusobacteriota showed no significant differences across groups (Figure 2b, Table S5). At the genus level, the most abundant bacterial genera in the periodontal conditions were *Prevotella*, *Streptococcus*, *Neisseria*, *Veillonella*, *Schaalia* and *Haemophilus* (Figure S1b). The dominant archaea included *Haloarcula* and *Halobellus*, fungi included *Candida* and *Aspergillus* and viruses included *Roseolovirus* and *Moineauvirus* (Figure 2c).

**FIGURE 2:**
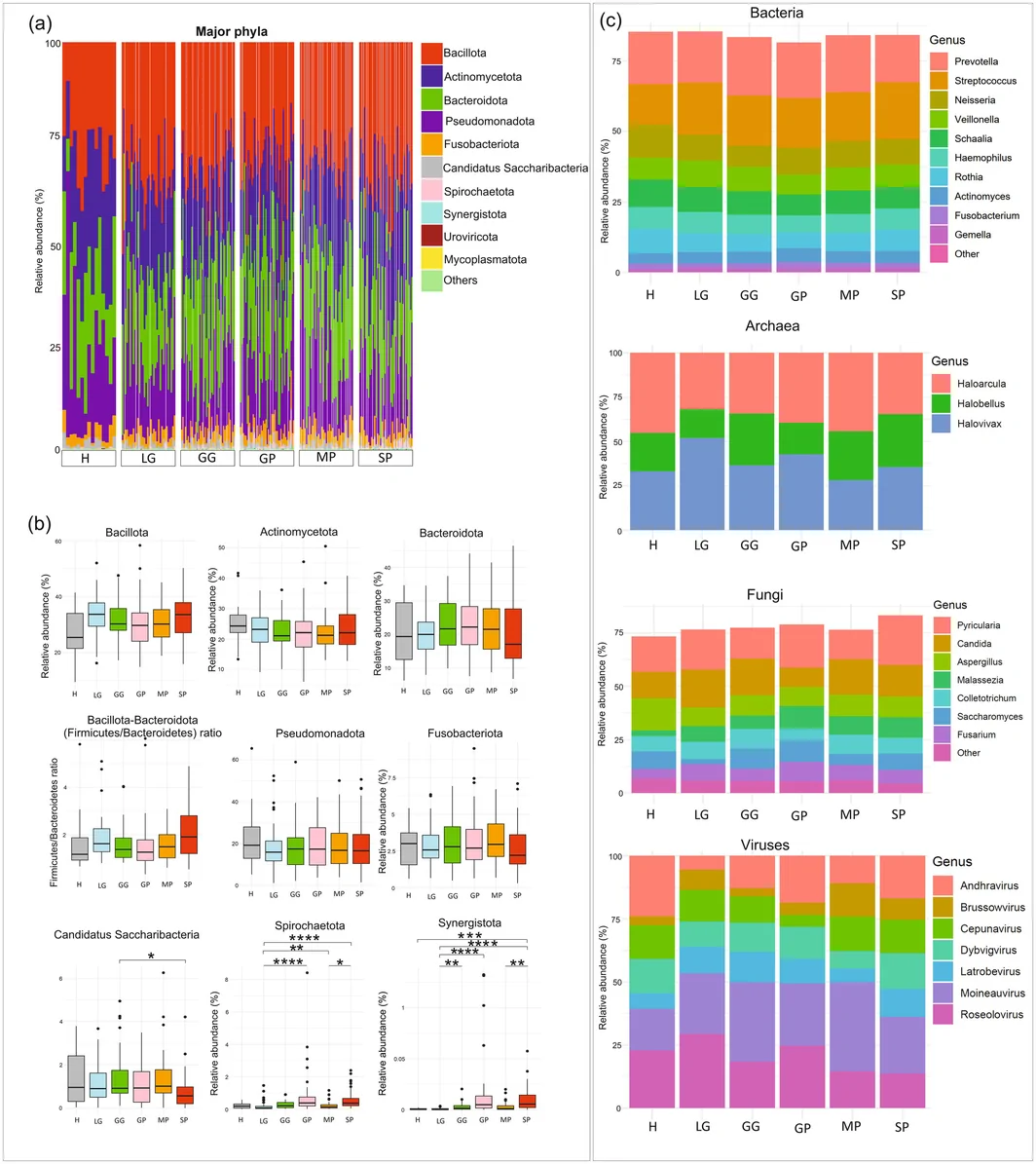
Differences in the abundance of major oral microbiome in different periodontal health and disease states. (a) Stacked bar plots showing sample‐wise relative abundance of the top 10 major phyla in different periodontal health and disease states. (b) Boxplots representing the mean relative abundance of major phyla detected in different periodontal health and disease states. *p < 0.05, **p < 0.01, ***p < 0.001, ****p < 0.0001 (Kruskal–Wallis test followed by Dunn's post‐test with Bonferroni correction). (c) Stacked bar plots showing mean relative abundance of microbial taxa at the genus levels within the bacterial, archaeal, fungal and viral kingdoms comparing individuals with different periodontal health and disease states. GG, generalised gingivitis; GP, gingivitis with pockets; H, periodontal health; LG, localised gingivitis; MP, mild periodontitis (Stage I/II); SP, severe periodontitis (Stage III/IV).

### 3.4 Microbial Taxa Associated With Periodontal Stages

LEfSe analysis identified distinct microbial species associated with each of the periodontal conditions (Figure 3; Table S6). When comparing H and LG, only *Lachnoanaerobaculum umeaense* was significantly more abundant in LG. Compared with H, GG showed a higher abundance of several early colonisers, including *Streptococcus oralis* , *Actinomyces oris* and *Prevotella veroralis* , whereas *Neisseria flavescens* was enriched in H. Compared with H, SP and GP showed significant enrichment of disease‐associated species, including the red complex ( *P. gingivalis* , *T. forsythia* , *T. denticola* ) and other pathogenic species such as *Porphyromonas endodontalis* , *Fusobacterium nucleatum* , *Prevotella denticola* , *S. oralis* , *Olsenella* sp. oral taxon 807 and *Parvimonas micra* .

**FIGURE 3:**
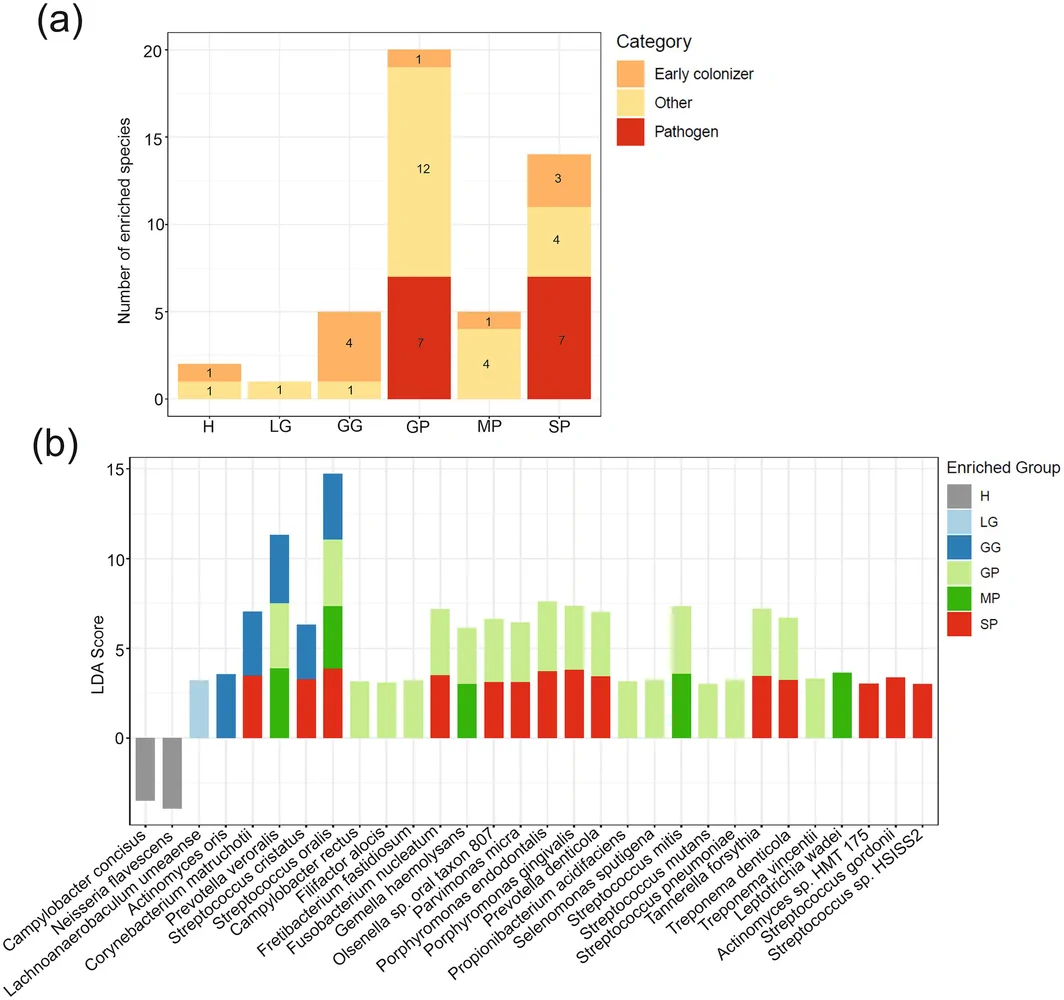
Differentially abundant oral microbial taxa across six periodontal conditions identified by LEfSe analysis. (a) Number of enriched oral microbial species across periodontal groups by category. (b) LDA scores of enriched oral microbial species across periodontal groups at the species level (LDA score > 3.0, *p* < 0.05). GG, generalised gingivitis; GP, gingivitis with pockets; H, periodontal health; LG, localised gingivitis; MP, mild periodontitis (Stage I–II); SP, severe periodontitis (Stage III–IV).

Correlation analysis showed that four species, including *Streptococcus mutans* and *Streptococcus sobrinus* , presented increasing trends from the H group to periodontitis (Figure 4a). In contrast, *P. gingivalis* , *T. denticola* , *T. forsythia* , *F. nucleatum* , *P. micra* and *P. endodontalis* showed higher abundance trends specifically in the GP and SP groups (Figure 4b). Conversely, three species, namely *Arcanobacterium haemolyticum* , *Bifidobacterium adolescentis* , and *Blautia pseudococcoides*, showed decreasing trends from H to SP (Figure 4c).

**FIGURE 4:**
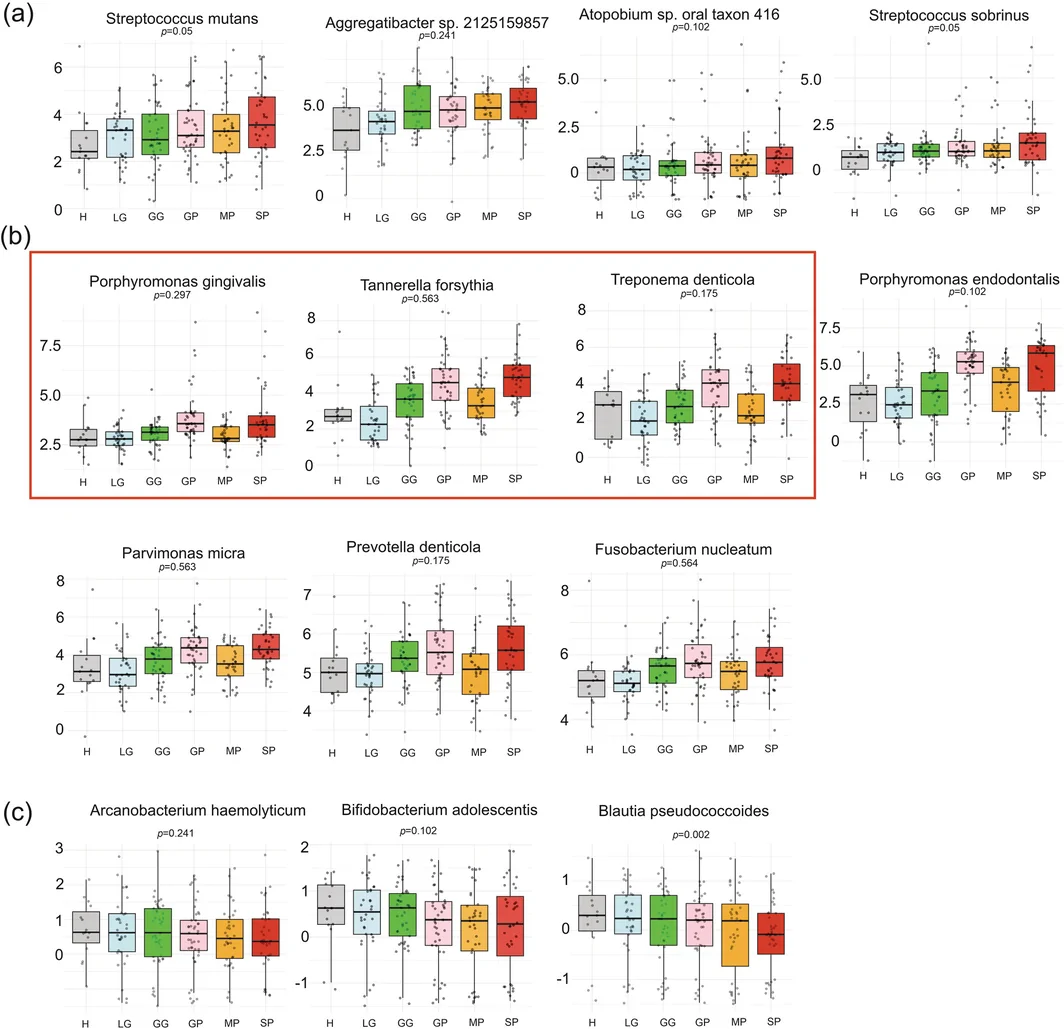
Species‐level monotonic abundance trends across periodontal groups. Boxplots showing CLR‐transformed relative abundances of microbial species across periodontal groups. Monotonic trends were assessed using Spearman rank correlation. (a) Species showing increasing trends from H to SP. (b) Species with higher abundances in GP and SP, including the red complex taxa ( *Porphyromonas gingivalis* , *Tannerella forsythia* and *Treponema denticola* ; highlighted by the red rectangle). (c) Species showing decreasing trends from H to SP. GG, generalised gingivitis; GP, gingivitis with pockets; H, periodontal health; LG, localised gingivitis; MP, mild periodontitis (Stage I–II); SP, severe periodontitis (Stage III–IV).

To distinguish GP and SP from H, we applied logistic regression to the abundances of nine significant periodontal species. The overall model achieved an AUC of 0.900 (95% CI: 0.770–1.000) for GP and 0.865 (95% CI: 0.762–0.968) for SP (Figure 5). Several key oral pathogens showed good discriminative performance in both groups. *T. forsythia* had the highest AUC in SP (0.900 [95% CI: 0.771–1.000]), whereas *P. endodontalis* showed the highest AUC in GP (0.861 [95% CI: 0.746–0.975]). *P. gingivalis* also showed good discrimination, with AUCs of 0.801 (95% CI: 0.653–0.949) in GP and 0.704 (95% CI: 0.546–0.862) in SP, indicating relevance in both groups but clearly stronger performance in GP. Most species showed moderate to high discriminative ability (AUC ≥ 0.70) in both groups (Table S7).

**FIGURE 5:**
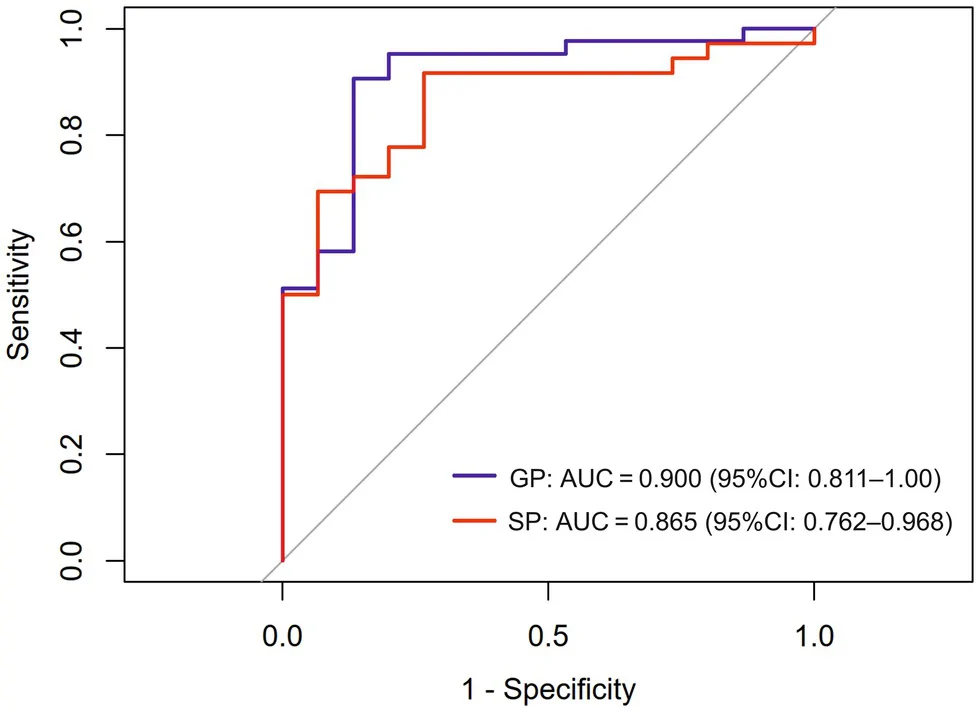
Receiver‐operating characteristic (ROC) curves distinguishing GP and SP from H subjects. ROC curves were generated using the combined abundance of nine key bacteria ( *Porphyromonas gingivalis* , *Tannerella forsythia* , *Treponema denticola* , *Porphyromonas endodontalis* , *Fusobacterium nucleatum* , *Prevotella denticola* , *Streptococcus oralis* , *Olsenella* sp. oral taxon 807 and *Parvimonas micra* ). Blue colour indicates comparisons between H and GP, and red colour indicates comparisons between H and SP.

### 3.5 Network Analysis

We constructed microbial networks to investigate co‐occurrence patterns in the oral microbiome across the periodontal conditions (Figure 6a). Co‐occurrence network analysis revealed distinct topological features across periodontal disease groups (Figure 6b, Table S8). SP exhibited the highest average degree, indicating a more connected network. In addition, the H group had a slightly larger number of edges. H and SP also had the highest network densities and largest number of clustering coefficients, suggesting tighter microbial co‐abundance associations (Figure 6c).

**FIGURE 6:**
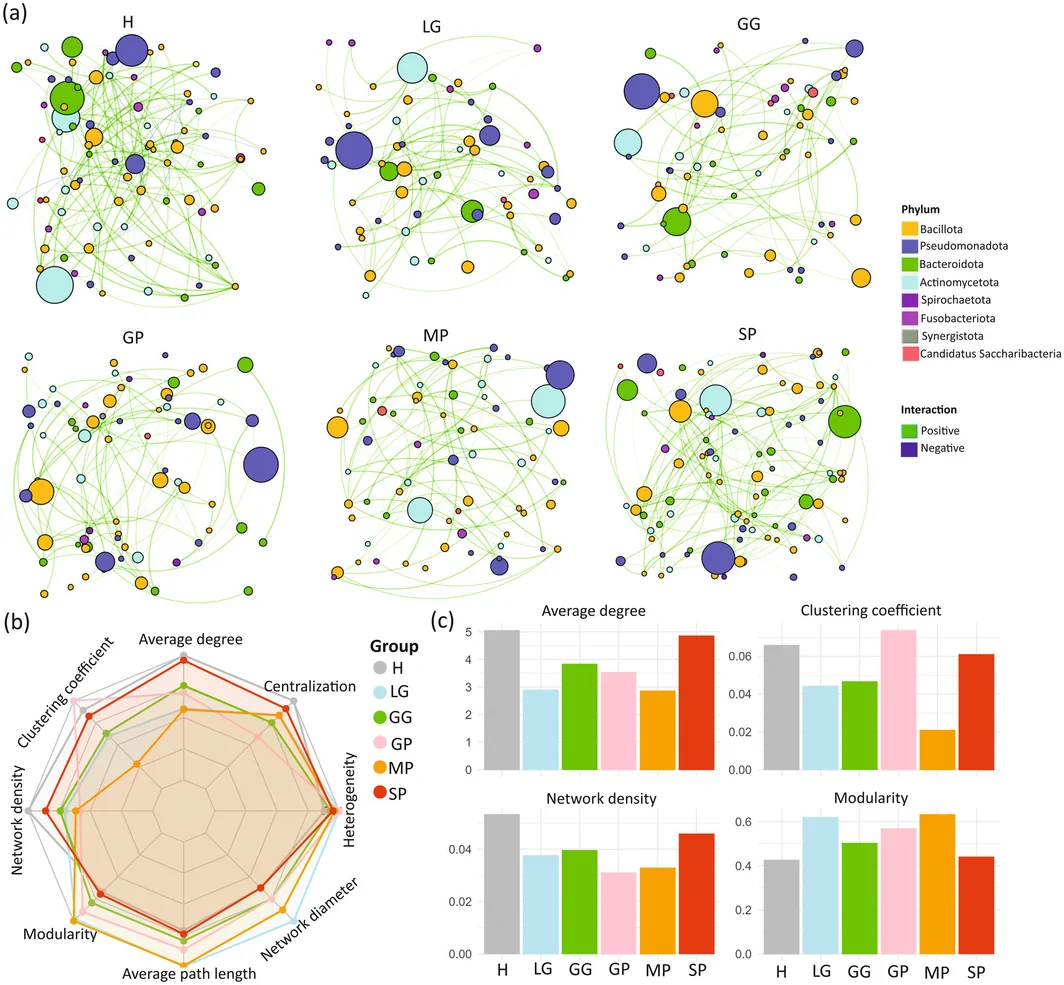
Microbial co‐occurrence network associated with periodontal health and disease states status. (a) Network graphs associated with periodontal health and disease states status. Each node represents a taxon at the species level. The size of the nodes is proportional to the mean relative abundance of taxa, and the colour of the nodes represents microbial taxa at the phylum level. The lines connecting the nodes represent a positive (green) or negative (blue) co‐occurrence relationship. (b) Radar plot showing key network topology metrics across groups. Each coloured polygon represents a different group's network, illustrating differences in connectivity, clustering, density, and community structure among groups. Higher values indicate greater intensity of the respective metric. (c) Boxplots showing the distribution of network metrics across groups. The plots illustrate variation and central tendency of these metrics within each group, highlighting differences in network topology. GG, generalised gingivitis; GP, gingivitis with pockets; H, periodontal health; LG, localised gingivitis; MP, mild periodontitis (Stage I/II); SP, severe periodontitis (Stage III/IV).

### 3.6 Differential Functional Pathways Across Periodontal Health and Disease States

Functional profiling identified 11 pathways that differed between the periodontal groups. Compared with H, LG showed an increase in PWY‐1042 (Glycolysis IV). In GG, 10 pathways were altered, including amino acid biosynthesis (SER‐GLYSYN‐PWY, VALSYN‐PWY [ *Corynebacterium matruchotii*]), nucleotide degradation (PWY0‐1296 [ *Streptococcus salivarius*]) and peptidoglycan biosynthesis (PWY‐6385 [*Leptotrichia* sp.]; *p* > 0.05). No significant functional shifts were observed in GP, MP or SP relative to H (Table S9).

## 4 Discussion

In this study, shotgun metagenomics uncovered the multi‐kingdom characteristics of the salivary microbiome across different periodontal conditions. Microbial diversity indices showed an increasing trend from periodontal health to gingivitis, followed by a decline in more advanced disease states. In addition to red complex bacteria, several species were associated with severe disease and appeared in substantial relative abundances when GG progressed to GP. A microbiome‐based model showed high, clinically relevant diagnostic accuracy, indicating that shifts in salivary bacteria closely reflect clinical progression.

Consistent with previous studies (Abusleme et al. 2021; Lu et al. 2021; Relvas et al. 2021; Iniesta et al. 2023), which reported lower microbial diversity in periodontal health than in disease stages, our saliva‐based findings support the utility of salivary profiles in capturing disease‐associated microbial shifts. Using the Shannon index, we observed a trend of increasing oral microbiome diversity from H to GP, followed by a decline in more advanced stages. In contrast, Inverse Simpson diversity remained relatively stable across the groups, with no statistically significant differences. This indicates that the observed alpha diversity changes are primarily driven by shifts in the abundance of less dominant taxa captured by Shannon diversity. Increasing diversity with formation of deepened periodontal pocketing is probably due to changes in the microbial environment.

In the present study, the six periodontal categories exhibited distinct microbial community compositions, as indicated by significant differences in both Bray–Curtis and Jaccard dissimilarity measures. Among the factors studied, only periodontal health status and smoking showed a strong association with beta diversity. Smoking, as previously reported in saliva‐based studies (Chopyk et al. 2021), influences the microbial community by shifting the abundance of disease‐associated taxa (Suzuki et al. 2022). Antibiotic use had some impact, whereas age and sex showed no effect, which is consistent with earlier findings (Park et al. 2015).

Gingival inflammation and pocketing (Loos and Van Dyke 2020) may initially increase the diversity by altering the niche and supporting disease‐associated species, which later gives way to pathogenic dominance as dysbiosis progresses. Generally, a deepened pocket of ≥ 4 mm is due to periodontal destruction (periodontal pocket) or may be due to chronic inflammation causing gingival enlargement with no bone loss (Bosshardt 2018). The present subjects in the GP group had increased pocket depths, which could be interpreted as pseudo‐pockets related to gingivitis because no alveolar bone loss was observed. The GP group represents an interesting and potential group of subjects immediately prior to the progression of gingivitis to periodontitis. Pocket formation in the GP group may reflect a highly inflammatory phenotype, in which an exaggerated soft‐tissue response leads to gingival enlargement without concurrent bone loss. Although immunological parameters were not assessed in the present cohort, such variability could have contributed to the delayed bone resorption observed in patients with gingivitis and periodontal pockets. Periodontal pockets, either true or pseudo, change the ecological environment, which increases bacterial count and favours certain species (Loos and Van Dyke 2020).

Many microbial taxa showed distinct profiles across the different periodontal states. The phyla Synergistota and Spirochaetota were more abundant in GP and SP, consistent with other studies reporting that these phyla are disease‐associated (Esparbes et al. 2021). Several genera, including *Streptococcus*, *Porphyromonas*, *Treponema*, *Tannerella*, *Olsenella* and *Parvimonas*, were significantly enriched in disease states compared with the periodontally healthy state. Although classical periodontal taxa, including *P. gingivalis* , *T. forsythia* and *T. denticola* , were enriched in the GP and SP groups, these species are not exclusive to the disease and may also occur in periodontal health. Increasing evidence suggests that such organisms function as pathobionts, whose impact depends on the microbial community context and host susceptibility rather than their mere presence. Recent studies have further demonstrated that oral pathobionts can contribute to systemic inflammatory conditions and evade host immune responses through multiple virulence mechanisms (Widziolek et al. 2025; Cömert et al. 2026). Therefore, our findings likely reflect ecological shifts and enrichment of disease‐associated communities, rather than disease stage–specific microbial signatures. This is consistent with studies showing substantial overlap in subgingival microbiome composition across periodontitis stages (Lafaurie et al. 2022), supporting the concept that disease progression is driven by polymicrobial dysbiosis rather than the appearance of unique pathogens. Our GP participants had eight or more sites with PPD ≥ 4 mm, which was above the average number in the MP group and similar to that in the SP group. Our findings are consistent with earlier reports linking these species to deeper periodontal sites and an enhanced inflammatory burden (Li et al. 2015; Scapoli et al. 2012).

Although our study found taxonomic differences in some non‐bacterial genera and species across the periodontal groups, none reached statistical significance. The dominant non‐bacterial genera included *Moineauvirus*, bacteriophages that primarily infect *Streptococcus* species and *Roseolovirus*, a member of the human herpesvirus family. Bacteriophages, the most abundant viruses in the oral cavity, can interact with oral microbial communities, modulate host responses, enhance bacterial virulence and facilitate horizontal gene transfer (Ambili et al. 2014). Among fungi, *Candida*, *Pyricularia* and *Aspergillus* were the most abundant genera. The oral mycobiome is diverse, with *Candida albicans* commonly detected and implicated in cross‐kingdom interactions that may influence periodontal inflammation (Diaz 2021; Hu et al. 2024). However, the low relative abundance of non‐bacterial taxa and the absence of significant differences between periodontal groups limit conclusions regarding their role in periodontal disease. Furthermore, environmental fungi such as *Aspergillus* and *Pyricularia* may reflect transient colonisation or environmental exposure rather than stable oral residents. Thus, our findings highlight the need to consider the oral microbiome as a multi‐kingdom ecosystem whose interactions may shape disease processes in ways not captured by taxonomic abundance alone.

Network analysis further supported stage‐specific ecological restructuring. The H group exhibited the highest network density and average degree, indicating dense and highly connected microbial interactions consistent with a stable and balanced community. Connectivity decreased in the intermediate disease stages, whereas the SP group again showed a more interconnected network, possibly reflecting stabilisation of a dysbiotic community dominated by disease‐associated taxa. Overall, these patterns suggest that microbial interaction networks gradually lose connectivity during early disease but regain a complex and densely connected structure in severe dysbiosis. Functional profiling revealed only modest metabolic shifts, primarily in gingivitis. In the GG group, alterations in amino acid biosynthesis, nucleotide degradation and peptidoglycan biosynthesis pathways suggested increased microbial turnover during early inflammation. The absence of significant functional differences between GP and periodontitis is consistent with previous observations of functional redundancy, whereby microbial communities maintain core metabolic functions despite substantial taxonomic variation (Li et al. 2014). In our cohort, this functional stability paralleled the relatively limited changes in functional diversity across disease stages.

Strengths of our study include a carefully planned design, detailed periodontal assessment and the use of shotgun metagenomic sequencing. Although the GP group was not included in the 2018 Classification of Periodontal Diseases (Chapple et al. 2018), it represented a clinically prominent cluster in our cohort and may reflect a transitional phase where gingivitis progresses towards periodontitis, potentially revealing microbiome differences. Limitations of the study include its cross‐sectional design, which prevented determining whether microbiome changes contributed to the shifts in periodontal status. Moreover, the absence of systematic CAL measurements may reduce diagnostic precision in transitional periodontal states such as gingivitis with pockets. However, radiographic bone levels and clinical findings support the absence of established periodontitis in these participants. While metagenomics better captures complex microbial shifts in periodontal disease, it also results in a superior ability with probable clinical relevance to differentiate both the early and severe stages of periodontitis. Although shotgun metagenomics allows multi‐kingdom profiling, non‐bacterial taxa were detected at a low relative abundance in this study. Our preliminary findings suggest that archaea, viruses and fungi may contribute to the overall oral microbial community structure; however, their clinical relevance in periodontal disease remains uncertain. Nevertheless, this study demonstrates the methodological advantage of shotgun metagenomics in capturing the full microbial landscape and highlights its potential to uncover clinically meaningful roles for microbial domains beyond bacteria as analytical resolution continues to improve. Although non‐bacterial taxa were detected at low abundance, their potential to shape microbial community dynamics and host responses suggests that future studies should consider periodontal diseases within a multi‐kingdom ecological framework rather than a purely bacterial paradigm.

## 5 Conclusion

Our study identified distinct salivary microbial community profiles across different periodontal health and disease stages. Microbial diversity and the relative abundance of several disease‐associated taxa increased from periodontal health to generalised gingivitis with gingival (pseudo) pockets and subsequently declined in periodontitis patients. These findings support the concept of periodontal disease as a process driven by polymicrobial dysbiosis rather than the presence of disease‐specific microorganisms. The observed multi‐kingdom microbial shifts provide insights into the transition from reversible gingivitis to irreversible periodontitis and may contribute to the identification of early indicators of disease progression.

## Author Contributions

Conceptualisation: M.M., E.K., P.J.P. and S.P. Acquisition and analysis of data for the work: M.M., J.L., E.K., J.P., P.J.P. and S.P. Interpretation of data for the work: M.M., J.L., E.K., L.L., J.P., P.J.P. and S.P. Writing and original draft preparation: M.M. and S.P. Revising the work critically: J.L., E.K., L.L., J.P. and P.J.P. Supervision: P.J.P. and S.P. Project administration: J.P. and S.P. Funding acquisition: J.P., P.J.P. and S.P. All authors have read and agreed with the final version of the manuscript.

## Funding

This work was supported by the Research Council of Finland grants (296541, 316777 and 355532 for S.P.; 340750 and 369310 for P.J.P.; and 286246, 318075, and 322656 for J.P.), the Finnish Dental Society Apollonia (for S.P. and P.J.P.), the Sigrid Juselius Foundation (for P.J.P. and J.P.) and the Helsinki and Uusimaa Hospital District (TYH2014407, TYH2018318 for J.P.).

## Ethics Statement

The study was approved by the local Ethics Committees and was conducted following Good Clinical Practice guidelines, the STROBE guidelines and the Declaration of Helsinki. All participants provided written informed consent.

## Conflicts of Interest

The authors declare no conflicts of interest.

## Supporting information


**Table S1:** Alpha diversity (Shannon and Inverse Simpson) of oral microbial differences across periodontal health and disease state groups.
**Table S2:** Pairwise comparison of alpha diversity (Shannon index) of oral microbial differences across periodontal health and disease state groups.
**Table S3:** Pairwise PERMANOVA (adonis2) results comparing microbial community composition at the species level based on Bray–Curtis and Jaccard distances.
**Table S4:** Adjusted PERMANOVA results for microbial community composition across periodontal groups, controlling for age, gender, smoking and recent antibiotic use.
**Table S5:** Relative abundance of major microbial taxa across periodontal health and disease states.
**Table S6:** List of differentially abundant microbial taxa across different taxonomic levels identified using LEfSe in periodontal health and disease groups. Only taxa with an LDA score > 3 are shown.
**Table S7:** Discriminative performance of key oral pathogens for GP and SP groups based on AUC and 95% confidence intervals.
**Table S8:** Comparison of topological features of co‐occurrence network properties between individuals with different periodontal health and disease states.
**Table S9:** Differentially abundant functional pathways across periodontal groups.
**Figure S1:** Pie chart showing the mean relative abundance of the top (a) phyla and (b) genera across all study groups. H, periodontal health; LG, localised gingivitis; GG, generalised gingivitis; GP, gingivitis with pockets; MP, mild periodontitis (Stage I/II); SP, severe periodontitis (Stage III/IV).

## Data Availability

The data that support the findings of this study are available in the European Genome‐Phenome Archive (accession no: EGAS00001007505). Metadata are not publicly available due to privacy restrictions but can be requested through the EGA under controlled access. The source code for the analyses is available at https://zenodo.org/records/15772816.

## References

[jcpe70184-bib-0001] Aas, J. A. , B. J. Paster , L. N. Stokes , I. Olsen , and F. E. Dewhirst . 2005. “Defining the Normal Bacterial Flora of the Oral Cavity.” Journal of Clinical Microbiology 43, no. 11: 5721–5732. 10.1128/JCM.43.11.5721-5732.2005. PMC1287824 16272510

[jcpe70184-bib-0002] Abusleme, L. , A. Dupuy , N. Dutzan , et al. 2013. “The Subgingival Microbiome in Health and Periodontitis and Its Relationship With Community Biomass and Inflammation.” ISME Journal 7: 1016–1025. 10.1038/ismej.2012.174. PMC3635234 23303375

[jcpe70184-bib-0003] Abusleme, L. , A. Hoare , and B. Y. Hong . 2021. “Microbial Signatures of Health, Gingivitis, and Periodontitis.” Periodontology 2000 86, no. 1: 57–78. 10.1111/prd.12362. 33690899

[jcpe70184-bib-0004] Ambili, R. , C. Preeja , V. Archana , et al. 2014. “Viruses: Are They Really Culprits for Periodontal Disease? A Critical Review.” Journal of Investigative and Clinical Dentistry 5, no. 3: 179–187. 10.1111/jicd.12029. 23447363

[jcpe70184-bib-0005] Aruni, A. W. , Y. Dou , A. Mishra , and H. M. Fletcher . 2015. “The Biofilm Community‐Rebels With a Cause.” Current Oral Health Reports 2, no. 1: 48–56. 10.1007/s40496-014-0044-5. PMC4478205 26120510

[jcpe70184-bib-0006] Bastian, M. , S. Heymann , and M. Jacomy . 2009. “Gephi: An Open Source Software for Exploring and Manipulating Networks.” Proceedings of the International AAAI Conference on Web and Social Media 3: 361–362.

[jcpe70184-bib-0007] Beghini, F. , L. J. McIver , A. Blanco‐Míguez , et al. 2021. “Integrating Taxonomic, Functional, and Strain‐Level Profiling of Diverse Microbial Communities With bioBakery 3.” eLife 10: e65088. 10.7554/eLife.65088. PMC8096432 33944776

[jcpe70184-bib-0008] Bosshardt, D. D. 2018. “The Periodontal Pocket: Pathogenesis, Histopathology and Consequences.” Periodontology 2000 76, no. 1: 43–50. 10.1111/prd.12153. 29194796

[jcpe70184-bib-0009] Cekici, A. , A. Kantarci , H. Hasturk , and T. E. van Dyke . 2014. “Inflammatory and Immune Pathways in the Pathogenesis of Periodontal Disease.” Periodontology 2000 64, no. 1: 57–80. 10.1111/prd.12002. PMC4500791 24320956

[jcpe70184-bib-0010] Chapple, I. L. C. , B. L. Mealey , T. E. Van Dyke , et al. 2018. “Periodontal Health and Gingival Diseases and Conditions on an Intact and a Reduced Periodontium: Consensus Report of Workgroup 1 of the 2017 World Workshop on the Classification of Periodontal and Peri‐Implant Diseases and Conditions.” Journal of Clinical Periodontology 89, no. Suppl 1: S74–S84. 10.1002/JPER.17-0719. 29926944

[jcpe70184-bib-0011] Chopyk, J. , C. M. Bojanowski , and J. Shin . 2021. “Compositional Differences in the Oral Microbiome of e‐Cigarette Users.” Frontiers in Microbiology 12: 599664. 10.3389/fmicb.2021.599664. PMC8200533 34135868

[jcpe70184-bib-0012] Cömert, F. , F. Yalçın , N. Topçuoğlu , et al. 2026. “Gestational Diabetes Alters Subgingival Pathobiont Composition.” Acta Odontologica Scandinavica 85: 179–189. 10.2340/aos.v85.45789. PMC13088773 41982206

[jcpe70184-bib-0013] Diaz, P. I. 2021. “Subgingival Fungi, Archaea, and Viruses Under the Omics Loupe.” Periodontology 2000 85, no. 1: 82–89. 10.1111/prd.12352. 33226731

[jcpe70184-bib-0014] Esparbes, P. , A. Legrand , and O. N. Bandiaky . 2021. “Subgingival Microbiota and Cytokines Profile Changes in Patients With Periodontitis: A Pilot Study Comparing Healthy and Diseased Sites in the Same Oral Cavities.” Microorganisms 9, no. 11: 2364. 10.3390/microorganisms9112364. PMC8618247 34835489

[jcpe70184-bib-0015] Genco, R. J. , and M. Sanz . 2020. “Clinical and Public Health Implications of Periodontal and Systemic Diseases: An Overview.” Periodontology 2000 83, no. 1: 7–13. 10.1111/prd.12344. 32385880

[jcpe70184-bib-0016] Hamp, S. E. , S. Nyman , and J. Lindhe . 1975. “Periodontal Treatment of Multirooted Teeth. Results After 5 Years.” Journal of Clinical Periodontology 2, no. 3: 126–135. 10.1111/j.1600-051x.1975.tb01734.x. 1058213

[jcpe70184-bib-0017] Hu, Y. , B. Ren , and L. Cheng . 2024. “Candida Species in Periodontitis: A New Villain or a New Target?” Journal of Dentistry 148: 105138. 10.1016/j.jdent.2024.105138. 38906455

[jcpe70184-bib-0018] Iniesta, M. , C. Chamorro , N. Ambrosio , M. J. Marín , M. Sanz , and D. Herrera . 2023. “Subgingival Microbiome in Periodontal Health, Gingivitis and Different Stages of Periodontitis.” Journal of Clinical Periodontology 50, no. 7: 905–920. 10.1111/jcpe.13793. 36792073

[jcpe70184-bib-0019] Jung, J.‐S. , J.‐K. Kook , S.‐N. Park , et al. 2024. “Salivary Microbiota Reflecting Changes in Subgingival Microbiota.” Microbiology Spectrum 12, no. 11: e0103024. 10.1128/spectrum.01030-24. PMC11537074 39365037

[jcpe70184-bib-0021] Kurgan, S. , and A. Kantarci . 2018. “Molecular Basis for Immunohistochemical and Inflammatory Changes During Progression of Gingivitis to Periodontitis.” Periodontology 2000 76, no. 1: 51–67. 10.1111/prd.12146. 29194785

[jcpe70184-bib-0022] Lafaurie, G. I. , Y. Neuta , R. Ríos , et al. 2022. “Differences in the Subgingival Microbiome According to Stage of Periodontitis: A Comparison of Two Geographic Regions.” PLoS One 17, no. 8: e0273523. 10.1371/journal.pone.0273523. PMC9398029 35998186

[jcpe70184-bib-0023] Lee, R. , J. Y. Park , Y. Park , K. Kim , J. W. Ha , and J. S. Lee . 2026. “Microbial Profiling of Saliva, Oral Rinse, Subgingival Plaque and GCF Reveals Site‐Specific Dysbiosis in Periodontitis: A Within‐Subject Comparison of 150 Participants.” Journal of Clinical Periodontology 53, no. 2: 179–189. 10.1111/jcpe.70050. PMC12803625 41122073

[jcpe70184-bib-0024] Lenartova, M. , B. Tesinska , T. Janatova , et al. 2021. “The Oral Microbiome in Periodontal Health.” Frontiers in Cellular and Infection Microbiology 11: 629723. 10.3389/fcimb.2021.629723. PMC8019927 33828997

[jcpe70184-bib-0025] Leskelä, J. , J. Putaala , N. Martinez‐Majander , et al. 2024. “Periodontitis, Dental Procedures, and Young‐Onset Cryptogenic Stroke.” Journal of Dental Research 103, no. 5: 494–501. 10.1177/00220345241232406. PMC11047014 38623924

[jcpe70184-bib-0026] Li, Y. , X. Feng , L. Xu , et al. 2015. “Oral Microbiome in Chinese Patients With Aggressive Periodontitis and Their Family Members.” Journal of Clinical Periodontology 42, no. 11: 1015–1023. 10.1111/jcpe.12463. 26412568

[jcpe70184-bib-0027] Li, Y. , J. He , Z. He , et al. 2014. “Phylogenetic and Functional Gene Structure Shifts of the Oral Microbiomes in Periodontitis Patients.” ISME Journal 8, no. 9: 1879–1891. 10.1038/ismej.2014.28. PMC4139721 24671083

[jcpe70184-bib-0028] Loos, B. G. , and T. E. Van Dyke . 2020. “The Role of Inflammation and Genetics in Periodontal Disease.” Periodontology 2000 83, no. 1: 26–39. 10.1111/prd.12297. PMC7319430 32385877

[jcpe70184-bib-0029] Lu, C. , Y. Chu , and J. R. Liu . 2021. “Subgingival Microbial Profiles of Young Chinese Adults With Stage I/II Periodontitis, Gingivitis and Periodontal Health Status.” Chinese Journal of Dental Research 24, no. 3: 167–175. 10.3290/j.cjdr.b1965003Mark. 34491011

[jcpe70184-bib-0030] Lu, J. , F. P. Breitwieser , P. Thielen , and S. L. Salzberg . 2017. “Bracken: Estimating Species Abundance in Metagenomics Data.” PeerJ Computer Science 3: e104. 10.7717/peerj-cs.104. PMC12016282 40271438

[jcpe70184-bib-0031] Mallick, H. , A. Rahnavard , L. J. McIver , et al. 2021. “Multivariable Association Discovery in Population‐Scale Meta‐Omics Studies.” PLoS Computational Biology 17, no. 11: e1009442. 10.1371/journal.pcbi.1009442. PMC8714082 34784344

[jcpe70184-bib-0032] Manzoor, M. , J. Leskelä , M. Pietiäinen , et al. 2024a. “Shotgun Metagenomic Analysis of the Oral Microbiome in Gingivitis: A Nested Case‐Control Study.” Journal of Oral Microbiology 16, no. 1: 2330867. 10.1080/20002297.2024.2330867. PMC10962305 38528961

[jcpe70184-bib-0033] Manzoor, M. , J. Leskelä , M. Pietiäinen , et al. 2024b. “Multikingdom Oral Microbiome Interactions in Early‐Onset Cryptogenic Ischemic Stroke.” ISME Communications 4, no. 1: ycae088. 10.1093/ismeco/ycae088. PMC11235082 38988699

[jcpe70184-bib-0034] Manzoor, M. , J. Putaala , S. Zaric , et al. 2025. “Oral Microbial Determinants of Saliva and Serum Lipopolysaccharide Activity.” Journal of Dental Research 105, no. 5: 578–586. 10.1177/00220345251370995. PMC13068149 41108124

[jcpe70184-bib-0035] Mohanty, R. , S. J. Asopa , M. D. Joseph , et al. 2019. “Red Complex: Polymicrobial Conglomerate in Oral Flora: A Review.” Journal of Family Medicine and Primary Care 8, no. 11: 3480–3486. 10.4103/jfmpc.jfmpc_759_19. PMC6881954 31803640

[jcpe70184-bib-0036] Nemoto, T. , T. Shiba , K. Komatsu , et al. 2021. “Discrimination of Bacterial Community Structures Among Healthy, Gingivitis, and Periodontitis Statuses Through Integrated Metatranscriptomic and Network Analyses.” mSystems 6, no. 6: e0088621. 10.1128/mSystems.00886-21. PMC8547322 34698525

[jcpe70184-bib-0037] Papapanou, P. N. , M. Sanz , N. Buduneli , et al. 2018. “Periodontitis: Consensus Report of Workgroup 2 of the 2017 World Workshop on the Classification of Periodontal and Peri‐Implant Diseases and Conditions.” Journal of Clinical Periodontology 89, no. Suppl 1: S173–S182. 10.1002/JPER.17-0721. 29926951

[jcpe70184-bib-0038] Park, O. J. , H. Yi , J. H. Jeon , et al. 2015. “Pyrosequencing Analysis of Subgingival Microbiota in Distinct Periodontal Conditions.” Journal of Dental Research 94, no. 7: 921–927. 10.1177/0022034515583531. 25904141

[jcpe70184-bib-0039] Pozhitkov, A. E. , B. G. Leroux , T. W. Randolph , T. Beikler , T. F. Flemmig , and P. A. Noble . 2015. “Towards Microbiome Transplant as a Therapy for Periodontitis: An Exploratory Study of Periodontitis Microbial Signature Contrasted by Oral Health, Caries and Edentulism.” BMC Oral Health 15: 125. 10.1186/s12903-015-0109-4. PMC4607249 26468081

[jcpe70184-bib-0040] Putaala, J. , N. Martinez‐Majander , S. Saeed , et al. 2017. “Searching for Explanations for Cryptogenic Stroke in the Young: Revealing the Triggers, Causes, and Outcome (SECRETO): Rationale and Design.” European Stroke Journal 2, no. 2: 116–125. 10.1177/2396987317703210. PMC6453214 31008307

[jcpe70184-bib-0041] Relvas, M. , A. Regueira‐Iglesias , C. Balsa‐Castro , et al. 2021. “Relationship Between Dental and Periodontal Health Status and the Salivary Microbiome: Bacterial Diversity, Co‐Occurrence Networks and Predictive Models.” Scientific Reports 11, no. 1: 929. 10.1038/s41598-020-79875-x. PMC7806737 33441710

[jcpe70184-bib-0042] Scapoli, L. , A. Girardi , A. Palmieri , et al. 2012. “Microflora and Periodontal Disease.” Dental Research Journal (Isfahan) 9, no. 2: S202‐6. 10.4103/1735-3327.109755. PMC3692174 23814584

[jcpe70184-bib-0043] Segata, N. , J. Izard , L. Waldron , et al. 2011. “Metagenomic Biomarker Discovery and Explanation.” Genome Biology 12: R60. 10.1186/gb-2011-12-6-r60. PMC3218848 21702898

[jcpe70184-bib-0044] Socransky, S. S. , A. D. Haffajee , M. A. Cugini , C. Smith , and R. L. Kent Jr. 1998. “Microbial Complexes in Subgingival Plaque.” Journal of Clinical Periodontology 25, no. 2: 134–144. 10.1111/j.1600-051x.1998.tb02419.x. 9495612

[jcpe70184-bib-0045] Suzuki, N. , Y. Nakano , M. Yoneda , T. Hirofuji , and T. Hanioka . 2022. “The Effects of Cigarette Smoking on the Salivary and Tongue Microbiome.” Cllinical and Experimental Dental Research 8, no. 1: 449–456. 10.1002/cre2.489. PMC8874080 34505401

[jcpe70184-bib-0046] Tonetti, M. S. , H. Greenwell , and K. S. Kornman . 2018. “Staging and Grading of Periodontitis: Framework and Proposal of a New Classification and Case Definition.” Journal of Periodontology 89, no. Suppl 1: S159‐S172. 10.1002/JPER.18-0006. 29926952

[jcpe70184-bib-0047] Tribble, G. D. , and R. J. Lamont . 2010. “Bacterial Invasion of Epithelial Cells and Spreading in Periodontal Tissue.” Periodontology 2000 52, no. 1: 68–83. 10.1111/j.1600-0757.2009.00323.x. PMC3647226 20017796

[jcpe70184-bib-0048] Widziolek, M. , A. Mieszkowska , M. Marcinkowska , et al. 2025. “Gingipains Protect *Porphyromonas gingivalis* From Macrophage‐Mediated Phagocytic Clearance.” PLoS Pathogens 21, no. 1: e1012821. 10.1371/journal.ppat.1012821. PMC11801703 39836688

[jcpe70184-bib-0049] Wood, D. E. , J. Lu , B. Langmead , et al. 2019. “Improved Metagenomic Analysis With Kraken 2.” Genome Biology 20, no. 1: 257. 10.1186/s13059-019-1891-0. PMC6883579 31779668

