# Supplementary material for: Shotgun Metagenomic Analysis Reveals Taxonomic and Functional Transitions in the Salivary Microbiome During Periodontal Disease Progression
Source: J Clin Periodontol. 2026 Aug 9;53(11):1650–62. doi: 10.1111/jcpe.70184 (PMC13658051; doi:10.1111/jcpe.70184)
Supplement: Supplementary file 1 [file JCPE-53-1650-s001.docx]

**Supplemental files**

**Shotgun Metagenomic Analysis Reveals Taxonomic and Functional Transitions in the Salivary Microbiome During Periodontal Disease Progression**

Muhammed Manzoor^1^, Jaakko Leskelä^1^, Eija Könönen^2^, Leo Lahti^3^, Jukka Putaala^4^, Pirkko J. Pussinen^1,5^, Susanna Paju^1^

^1^ Department of Oral and Maxillofacial Diseases, University of Helsinki, Helsinki, Finland

^2^ Institute of Dentistry, University of Turku, Turku, Finland

^3^ Department of Computing, University of Turku, Turku, Finland

^4^ Department of Neurology, Helsinki University Hospital and University of Helsinki, Helsinki, Finland

^5^ School of Medicine, Institute of Dentistry, University of Eastern Finland, Kuopio, Finland

**Correspondence:** Muhammed Manzoor (muhammed.allippara@helsinki.fi)

**Supplementary Methods**

**Study Subjects and Recruitment**

The present study, SECRETO Oral **(Leskelä et al. 2024; Manzoor et al. 2024b)**, is a sub-study of the Search for Explanations for Cryptogenic Stroke in the Young: Revealing the Etiology and Triggers (SECRETO), an international, prospective, multicenter study **(Putaala et al. 2017)**. Participants were recruited from December 2013 to November 2019 at the Helsinki University Hospital and Turku University Central Hospital. The study was approved by the local Ethics Committees and conducted following the Good Clinical Practice guidelines and the Declaration of Helsinki. All the participants provided written informed consent. Participants were recruited from December 2013 to November 2019 at the Helsinki University Hospital and Turku University Central Hospital.

The study enrolled adults aged 18–49 years who underwent a comprehensive structured interview upon recruitment, and detailed clinical histories were obtained from their medical records. This included data on antibiotic use, hypertension, smoking, obesity, diabetes mellitus, current or former tobacco use, heavy alcohol consumption, and any illicit drug use.

**Clinical oral examination**

The clinical parameters of the study participants were assessed by a single periodontist (S.P.) between April 2014 and February 2020. Panoramic tomography images were obtained for all participants.

Clinical moderate or extensive caries lesions were diagnosed and classified using the International Caries Detection and Assessment System (ICDAS). Four subjects had dental implants, and their peri-implant status was in accordance with their overall periodontal status.

**Saliva Sampling and Metagenomic Sequencing**

Sample processing and DNA extraction are described in detail elsewhere **(Manzoor et al. 2024b; 2025**). Briefly, participants chewed paraffin to stimulate at least 4 mL of saliva, which was collected in RNase/DNase-free tubes and stored at −70 °C. Genomic DNA was extracted and metagenomic sequencing was performed on an Illumina NovaSeq 6000. Taxonomy was assigned using Kraken2 (v.2.1.2**) (Wood et al. 2019)**, and Bracken (v.2.7.0) **(Lu et al. 2017).**

**Statistical Analysis**

Statistical analyses were conducted using R (v. 4.2.2). Summary statistics were reported as means ± SD for continuous variables and counts (%) for categorical variables. Group differences were assessed using ANOVA or Kruskal-Wallis tests for continuous variables, and χ² or Fisher’s exact tests for categorical variables.

Prior to statistical analyses, microbiome data were filtered to retain taxa present in at least 10% of samples with a relative abundance greater than 0.001%. Microbial alpha diversity was assessed using the Shannon and Inverse Simpson indices, calculated from filtered species-level count data using the *microbiome* (v1.20.0) and *vegan* packages. Normality was evaluated using the Shapiro-Wilk test. Based on distribution characteristics, one-way ANOVA followed by Tukey’s HSD post-hoc test was used for Shannon diversity, while non-parametric Dunn’s tests were applied to compare Inverse Simpson indices across groups. Permutational multivariate analysis of variance (PERMANOVA) was conducted using Bray-Curtis dissimilarity and Jaccard distance metrics to assess differences in microbial community composition across periodontal groups, adjusting for age, sex, smoking history, and recent antibiotic use. Principal coordinates analysis (PCoA) was applied to both distance matrices to visualize species-level beta diversity differences among groups. Top abundant taxa were selected by cumulative abundance, and group-wise mean relative abundances per sample were visualized with boxplots. Differential abundance analysis was performed using LEfSe (Linear Discriminant Analysis Effect Size; **Segata et al. 2011**). Statistical significance between periodontal groups was assessed using Kruskal-Wallis and Wilcoxon tests (*p* < 0.05). Only taxa with LDA scores >3 were considered significant. To test monotonic trends of microbial taxa across periodontal groups, we used Spearman rank correlation.

To explore the characteristics of oral microbiomes in GP and SP patients as compared to H subjects, a Lasso logistic regression model was fitted with five-fold cross-validation to identify discriminant species based on the area under the receiver operating characteristic (ROC) curve (AUC). Model performance was assessed using ROC analysis. For network analysis, a SparCC-based co-occurrence network was built using the trans_network class with SpiecEasi and a 0.001 abundance filter using the *microeco* (v. 1.7.1) R package. Network visualization was done with Gephi (v0.9.7) **(Bastian et al. 2009).** Differential abundance *p*-values were adjusted for multiple testing using the Benjamini–Hochberg false discovery rate (FDR) procedure.

Supplementary Sources

Bastian, M., Heymann, S., Jacomy, M. 2009. Gephi: an open source software for exploring and manipulating networks. *Proceedings of the International AAAI Conference on Web and Social Media*. 3, no. 361-362.

Leskelä, J., Putaala, J., Martinez-Majander, N., et al. 2024. Periodontitis, dental procedures, and young-onset cryptogenic stroke. *Journal of Dental Research*. 103(5), no. 494-501. doi: 10.1177/00220345241232406. PMC11047014.

Manzoor, M., Leskelä, J., Pietiäinen, M. et al. 2024. Shotgun metagenomic analysis of the oral microbiome in gingivitis: a nested case-control study. *Journal of Oral Microbiology* . 16(1), no. 2330867. doi: 10.1080/20002297.2024.2330867.

Putaala, J., Martinez-Majander, N., Saeed, S., et al. 2017. Searching for explanations for cryptogenic stroke in the young: Revealing the triggers, causes, and outcome (SECRETO): Rationale and design. *Eur Stroke J*. 2(2), no. 116-125. doi: 10.1177/2396987317703210.

Segata, N., Izard, J., Waldron, L. et al. 2011. Metagenomic biomarker discovery and explanation. *Genome Biology* 12, no. R60 https://doi.org/10.1186/gb-2011-12-6-r60

**Table S1.** Alpha diversity (Shannon and Inverse Simpson) of oral microbial differences across periodontal health and disease state groups.

|  | **H** | **LG** | **GG** | **GP** | **MP** | **SP** | ***P* value** |
| --- | --- | --- | --- | --- | --- | --- | --- |
| Shannon | 3.90 (0.281) | 4.032 (0.202 | 4.072 (0.248) | 4.151 (0.275) | 4.088 (0.210) | 4.051 (0.213) | 0.0183 * |
| Inverse Simpson | 20.534 (7.159) | 24.044 (5.706) | 24.551 (6.128) | 27.095 (9.427) | 25.211 (6.849) | 24.060 (6.083) | 0.156 |

H: Periodontal Health, LG: Localized gingivitis, GG: Generalized gingivitis, GP: gingivitis with pockets, MP: Mild periodontitis (Stage I/II), SP: Severe periodontitis (Stage III/IV)

**Table S2.** Pairwise comparison of alpha diversity (Shannon index) of oral microbial differences across periodontal health and disease state groups

| **Group** | **Difference (diff)** | **Lower CI (lwr)** | **Upper CI (upr)** | ***P* value** |
| --- | --- | --- | --- | --- |
| H vs LG | 0.1321 | -0.0799 | 0.3442 | 0.4725 |
| H vs GG | 0.1729 | -0.0336 | 0.3793 | 0.1578 |
| H vs GP | 0.2511 | 0.0460 | 0.4563 | **0.0069*** |
| H vs MP | 0.1887 | -0.0224 | 0.3999 | 0.1089 |
| H vs SP | 0.1518 | -0.0585 | 0.3620 | 0.3035 |
| LG vs GG | 0.0407 | -0.1180 | 0.1994 | 0.9768 |
| LG vs GP | 0.1190 | -0.0380 | 0.2760 | 0.2513 |
| LG vs MP | 0.0566 | -0.1081 | 0.2213 | 0.9210 |
| LG vs SP | 0.0196 | -0.1440 | 0.1832 | 0.9993 |
| GG vs GP | 0.0783 | -0.0711 | 0.2276 | 0.6595 |
| GG vs MP | 0.0159 | -0.1416 | 0.1733 | 0.9997 |
| GG vs SP | -0.0211 | -0.1774 | 0.1351 | 0.9988 |
| GP vs MP | -0.0624 | -0.2181 | 0.0934 | 0.8585 |
| GP vs SP | -0.0994 | -0.2539 | 0.0552 | 0.4361 |
| MP vs SP | -0.0370 | -0.1994 | 0.1254 | 0.9864 |

H: Periodontal Health, LG: Localized gingivitis, GG: Generalized gingivitis, GP: gingivitis with pockets, MP: Mild periodontitis (Stage I/II), SP: Severe periodontitis (Stage III/IV)

**Table S3.** Pairwise PERMANOVA (adonis2) results comparing microbial community composition at the species level based on Bray–Curtis and Jaccard distances

|  | **Bray–Curtis** | | | **Jaccard** | | |
| --- | --- | --- | --- | --- | --- | --- |
| **Group** | **R^2^** | **F value** | ***P* value** | **R^2^** | **F value** | ***P* value** |
| H vs LG | 0.013 | 0.622 | 0.850 | 0.017 | 0.825 | 0.696 |
| H vs GG | 0.019 | 1.085 | 0.331 | 0.019 | 1.109 | 0.289 |
| H vs GP | 0.025 | 1.506 | 0.118 | 0.022 | 1.263 | 0.196 |
| H vs MP | 0.017 | 0.816 | 0.573 | 0.018 | 0.879 | 0.566 |
| H vs SP | 0.021 | 1.071 | 0.333 | 0.022 | 1.091 | 0.0297 |
| LG vs GG | 0.014 | 1.039 | 0.337 | 0.015 | 1.105 | 0.291 |
| LG vs GP | 0.033 | 2.636 | **0.004**** | 0.027 | 2.101 | **0.011*** |
| LG vs MP | 0.010 | 0.683 | 0.736 | 0.012 | 0.798 | 0.703 |
| LG vs SP | 0.026 | 1.797 | 0.072 | 0.025 | 1.737 | **0.037*** |
| GG vs GP | 0.018 | 1.506 | 0.118 | 0.017 | 1.433 | 0.097 |
| GG vs MP | 0.007 | 0.494 | 0.929 | 0.008 | 0.661 | 0.886 |
| GG vs SP | 0.027 | 1.816 | 0.069 | 0.022 | 1.699 | 0.057 |
| GP vs MP | 0.019 | 1.481 | 0.134 | 0.016 | 1.250 | 0.152 |
| GP vs SP | 0.016 | 1.221 | 0.233 | 0.015 | 1.209 | 0.200 |
| MP vs SP | 0.022 | 1.553 | 0.124 | 0.021 | 1.478 | 0.083 |

H: Periodontal Health, LG: Localized gingivitis, GG: Generalized gingivitis, GP: gingivitis with pockets, MP: Mild periodontitis (Stage I/II), SP: Severe periodontitis (Stage III/IV)

**Table S4.** Adjusted PERMANOVA results for microbial community composition across periodontal groups, controlling for age, gender, smoking, and recent antibiotic use.

|  | **Bray–Curtis** | | | **Jaccard** | | |
| --- | --- | --- | --- | --- | --- | --- |
| **Group** | **R^2^** | **F value** | ***P* value** | **R^2^** | **F value** | ***P* value** |
| Periodontal groups | 0.031 | 1.279 | **0.048** | 0.312 | 1.279 | 0.053 |
| Age | 0.003 | 0.667 | 0.894 | 0.003 | 0.667 | 0.879 |
| Sex | 0.003 | 0.702 | 0.839 | 0.003 | 0.702 | 0.848 |
| Smoking ever | 0.008 | 1.663 | **0.042*** | 0.008 | 1.663 | **0.044*** |
| Antibiotics (preceding 3 months) | 0.007 | 1.445 | 0.080 | 0.007 | 1.445 | 0.119 |

**Table S5.** Relative abundance of major microbial taxa across periodontal health and disease states

| **Taxon** | **H** | **LG** | **GG** | **GP** | **MP** | **SP** | ***p*-value** |
| --- | --- | --- | --- | --- | --- | --- | --- |
| Bacillota | 26.81 | 33.18 | 31.63 | 29.82 | 30.59 | 33.18 | **0.048** |
| Actinomycetota | 25.07 | 23.04 | 22.37 | 22.20 | 22.61 | 23.11 | 0.759 |
| Bacteroidota | 20.78 | 19.87 | 22.70 | 23.50 | 22.38 | 20.10 | 0.294 |
| Pseudomonadota | 22.67 | 19.49 | 18.32 | 18.91 | 19.23 | 19.22 | 0.944 |
| Fusobacteriota | 2.88 | 2.85 | 3.06 | 3.24 | 3.33 | 2.66 | 0.490 |
| Candidatus Saccharibacteria | 1.36 | 1.17 | 1.41 | 1.11 | 1.43 | 0.77 | **0.036** |
| Spirochaetota | 0.215 | 0.212 | 0.263 | 0.879 | 0.241 | 0.632 | **<0.001** |
| Synergistota | 0.008 | 0.007 | 0.030 | 0.158 | 0.031 | 0.107 | **<0.001** |
| Uroviricota | 0.043 | 0.038 | 0.040 | 0.043 | 0.038 | 0.045 | 0.416 |
| Mycoplasmatota | 0.005 | 0.009 | 0.027 | 0.015 | 0.012 | 0.015 | **0.009** |
| Bacillota/ Bacteroidota (Firmicutes/Bacteroidetes) ratio | 1.700 | 1.903 | 1.586 | 1.528 | 1.582 | 2.111 | **0.038** |

H: Periodontal health, LG: Localized gingivitis, GG: Generalized gingivitis, GP: gingivitis with pockets, MP: Mild periodontitis (Stage I/II), SP: Severe periodontitis (Stage III/IV)

**Table S6.** List of differentially abundant microbial taxa across different taxonomic levels identified using LEfSe in periodontal health and disease groups. Only taxa with an LDA score >3 are shown.

| **Comparison** | **Feature** | **Enriched Group** | **LDA Score** | **P-value** |
| --- | --- | --- | --- | --- |
| **H vs LG** | *Lachnoanaerobaculum umeaense* | LG | 3.20 | 0.041 |
| **H vs GG** | *Neisseria flavescens* | H | -3.92 | 0.047 |
|  | *Prevotella veroralis* | GG | 3.79 | 0.014 |
|  | *Streptococcus oralis* | GG | 3.66 | 0.004 |
|  | *Corynebacterium matruchotii* | GG | 3.56 | 0.021 |
|  | *Actinomyces oris* | GG | 3.55 | 0.049 |
|  | *Streptococcus cristatus* | GG | 3.05 | <0.001 |
| **H vs GP** | *Porphyromonas endodontalis* | GP | 3.88 | <0.001 |
|  | *Streptococcus mitis* | GP | 3.77 | 0.018 |
|  | *Tannerella forsythia* | GP | 3.76 | <0.001 |
|  | *Streptococcus oralis* | GP | 3.71 | 0.008 |
|  | *Fusobacterium nucleatum* | GP | 3.69 | 0.003 |
|  | *Prevotella veroralis* | GP | 3.64 | 0.002 |
|  | *Prevotella denticola* | GP | 3.58 | 0.015 |
|  | *Porphyromonas gingivalis* | GP | 3.56 | 0.002 |
|  | *Olsenella* sp. oral taxon 807 | GP | 3.54 | 0.017 |
|  | *Treponema denticola* | GP | 3.47 | 0.011 |
|  | *Parvimonas micra* | GP | 3.31 | 0.003 |
|  | *Treponema vincentii* | GP | 3.30 | 0.009 |
|  | *Selenomonas sputigena* | GP | 3.24 | 0.008 |
|  | *Streptococcus pneumoniae* | GP | 3.24 | 0.046 |
|  | *Fretibacterium fastidiosum* | GP | 3.21 | <0.001 |
|  | *Propionibacterium acidifaciens* | GP | 3.15 | 0.035 |
|  | *Campylobacter rectus* | GP | 3.14 | 0.004 |
|  | *Gemella haemolysans* | GP | 3.13 | 0.006 |
|  | *Filifactor alocis* | GP | 3.09 | <0.001 |
|  | *Streptococcus mutans* | GP | 3.02 | 0.037 |
| **H vs MP** | *Prevotella veroralis* | MP | 3.88 | 0.011 |
|  | *Leptotrichia wadei* | MP | 3.64 | 0.010 |
|  | *Streptococcus mitis* | MP | 3.58 | 0.043 |
|  | *Streptococcus oralis* | MP | 3.50 | 0.002 |
|  | *Gemella haemolysans* | MP | 3.00 | 0.006 |
| **H vs SP** | *Campylobacter concisus* | H | -3.49 | 0.032 |
|  | *Streptococcus oralis* | SP | 3.85 | <0.001 |
|  | *Porphyromonas gingivalis* | SP | 3.80 | 0.021 |
|  | *Porphyromonas endodontalis* | SP | 3.72 | <0.001 |
|  | *Fusobacterium nucleatum* | SP | 3.49 | 0.009 |
|  | *Corynebacterium matruchotii* | SP | 3.47 | 0.026 |
|  | *Tannerella forsythia* | SP | 3.45 | <0.001 |
|  | *Prevotella denticola* | SP | 3.44 | 0.035 |
|  | *Streptococcus gordonii* | SP | 3.37 | <0.001 |
|  | *Streptococcus cristatus* | SP | 3.26 | <0.001 |
|  | *Treponema denticola* | SP | 3.24 | 0.008 |
|  | *Parvimonas micra* | SP | 3.12 | 0.003 |
|  | *Olsenella* sp. oral taxon 807 | SP | 3.11 | 0.014 |
|  | *Actinomyces* sp. HMT 175 | SP | 3.03 | 0.033 |
|  | *Streptococcus* sp. HSISS2 | SP | 3.01 | 0.012 |

**Table S7.** Discriminative performance of key oral pathogens for GP and SP groups based on AUC and 95% confidence intervals

| **Species** | **AUC (GP) [95% CI]** | **AUC (SP) [95% CI]** |
| --- | --- | --- |
| *P. gingivalis* | 0.8008 [0.6528–0.9488] | 0.7037 [0.5459–0.8615] |
| *T. forsythia* | 0.8008 [0.6646–0.9370] | 0.9000 [0.7705–1.0000] |
| *T.denticola* | 0.7147 [0.5704–0.8591] | 0.6843 [0.5333–0.8352] |
| *P.endodontalis* | 0.8605 [0.7462–0.9747] | 0.8009 [0.6777–0.9241] |
| *F.nucleatum* | 0.7488 [0.5929–0.9047] | 0.7231 [0.5596–0.8866] |
| *P. denticola* | 0.7271 [0.5883–0.8659] | 0.7435 [0.6021–0.8849] |
| *S. oralis* | 0.7248 [0.5819–0.8677] | 0.8130 [0.6796–0.9463] |
| *Olsenella* sp. oral taxon 807 | 0.7202 [0.5790–0.8613] | 0.7148 [0.5545–0.8751] |
| *Parvimonas micra* | 0.7395 [0.5809–0.8982] | 0.7528 [0.5913–0.9143] |

**Table S8.** Comparison of topological features of co-occurrence network properties between individuals with different periodontal health and disease states. Networks were constructed using Spearman correlation, with an abundance filter of 0.001, a correlation coefficient threshold of 0.7, and a *p*-value threshold of 0.01.

|  | **H** | **LG** | **GG** | **GP** | **MP** | **SP** |
| --- | --- | --- | --- | --- | --- | --- |
| No of features | 99 | 91 | 107 | 119 | 103 | 112 |
| Vertex | 96.0 | 78.0 | 98 | 115 | 88.0 | 107.0 |
| Edge | 243.00 | 113.0 | 188 | 203 | 126.00 | 260.0 |
| Average degree | 5.06 | 2.89 | 3.83 | 3.53 | 2.863 | 4.859 |
| Average path length | 2.350 | 3.301 | 2.623 | 2.85 | 3.283 | 2.441 |
| Network diameter | 5.000 | 8.00 | 6.0 | 6.00 | 7.0 | 5.00 |
| Clustering coefficient | 0.0658 | 0.0443 | 0.0466 | 0.073 | 0.0208 | 0.061 |
| Network density | 0.0532 | 0.0376 | 0.039 | 0.030 | 0.0329 | 0.045 |
| Heterogeneity | 0.6787 | 0.7699 | 0.7166 | 0.7701 | 0.741 | 0.7313 |
| Centralization | 0.1256 | 0.1052 | 0.0945 | 0.0743 | 0.105 | 0.1145 |
| Modularity | 0.42597 | 0.6205 | 0.5034 | 0.568 | 0.6329 | 0.4407 |

H: Periodontal health, LG: Localized gingivitis, GG: Generalized gingivitis, GP: gingivitis with pockets, MP: Mild periodontitis (Stage I/II), SP: Severe periodontitis (Stage III/IV)

**Table S9.** Differentially abundant functional pathways across periodontal groups.

| **Comparison** | **Pathway (Taxon)** | **Coef** | **SE** | **p-value** | **q-value** |
| --- | --- | --- | --- | --- | --- |
| HL vs LG | PWY-1042 Glycolysis IV (*Streptococcus vestibularis*) | 0.008 | 0.002 | <0.001 | 0.078 |
| HL vs GG | PWY-6147 6-hydroxymethyl-dihydropterin diphosphate biosynthesis I (*Neisseria subflava*) | -0.004 | 0.001 | <0.001 | 0.287 |
| HL vs GG | PWY-6163 Chorismate biosynthesis (*Prevotella melaninogenica*) | 0.005 | 0.001 | <0.001 | 0.287 |
| HL vs GG | PWY-6385 Peptidoglycan biosynthesis III (*Leptotrichia* sp. oral taxon 215) | -0.002 | 0.001 | <0.001 | 0.287 |
| HL vs GG | PWY-7221 Guanosine ribonucleotides de novo biosynthesis (*Leptotrichia* sp. oral taxon 215) | -0.003 | 0.001 | <0.001 | 0.287 |
| HL vs GG | PWY-7953 UDP-N-acetylmuramoyl-pentapeptide biosynthesis III (*Veillonella parvula*) | 0.003 | 0.001 | <0.001 | 0.287 |
| HL vs GG | PWY0-1296 Purine ribonucleosides degradation (*Streptococcus salivarius*) | 0.006 | 0.002 | <0.001 | 0.287 |
| HL vs GG | SER-GLYSYN-PWY L-serine and glycine biosynthesis I (*Corynebacterium matruchotii*) | 0.004 | 0.001 | <0.001 | 0.287 |
| HL vs GG | UDPNAGSYN-PWY UDP-N-acetyl-D-glucosamine biosynthesis I (*Corynebacterium matruchotii*) | 0.004 | 0.001 | <0.001 | 0.287 |
| HL vs GG | VALSYN-PWY L-valine biosynthesis (*Corynebacterium matruchotii*) | 0.005 | 0.001 | <0.001 | 0.287 |
| HL vs GG | PWY-1042 Glycolysis IV (*Streptococcus vestibularis*) | 0.006 | 0.002 | <0.001 | 0.299 |

**Figure S1.** Pie chart showing the mean relative abundance of the top (a) phyla and (b) genera across all study groups. H, periodontal health; LG, localized gingivitis; GG, generalized gingivitis; GP, gingivitis with pockets; MP, mild periodontitis (Stage I/II); SP, severe periodontitis (Stage III/IV).
